# The influence of physical activity during sleep deprivation on mood and reaction speed to visual and auditory stimuli

**DOI:** 10.1192/j.eurpsy.2023.893

**Published:** 2023-07-19

**Authors:** M. Sochal, M. Ditmer, S. Turkiewicz, F. Karuga, D. Strzelecki, A. Gabryelska

**Affiliations:** 1 Department of Sleep Medicine and Metabolic Disorders; 2 Department of Affective and Psychotic Disorders, Medical University of Lodz, Poland, Lodz, Poland

## Abstract

**Introduction:**

Sleep deprivation (SD) is being examined in the treatment of depression and other affective disorders for years. However, studies’ outcomes remain ambiguous, with varying levels of clinical improvement and its ephemeral character. Thus, it is necessary to find new factors accounting for the variability of results to develop new therapeutic protocols.

**Objectives:**

The study aimed to assess the influence of physical activity on mood and reaction speed following SD.

**Methods:**

The study group consisted of 71 participants. SD lasted about 24 hours, beginning in the morning hours of the SD day to the morning hours of the following day. Physical activity (PA) was controlled using actigraphy (actigraph GENEActive Original, ActivInsights Ltd.) given to each participant. Participants underwent the reaction speed test (Response Time Test Apparatus, AT Smart Systems, Poland) and filled out a questionnaire assessing depression symptoms- Beck Depression Inventory (BDI), in the evening of the SD day, and the following morning. Based on the percentage of sedentary time (gravity-subtracted sum of vector magnitudes<386, DOI 10.1111/sms.13488) participants were classified as inactive (≥70% of SD duration spent sedentary, n= 43) or active (n= 28).

**Results:**

There were no significant differences between the active and the inactive participants regarding pre/post SD BDI score, reaction speed, and demographic data (age, sex, BMI) (all p>0.05). The inactive group had a significantly lower BDI score following SD in comparison with their baseline parameters (5, IQR 1-12 vs. 3, IQR 0-12, p=0.024) than the active group (3, IQR 1-6 vs. 3, IQR 0-7, p=0.408). Reaction speed after SD was impaired in both active (0.216, IQR 0.206-0.226 vs. 0.231, IQR 0.222-0.46, p<0.001) and inactive group (0.224, IQR 0.216-0.235 vs. 0.238, IQR 0.220-0.251, p<0.001). However, the difference between pre/post SD response time was slightly higher in the active individuals (0.015, IQR 0.011-0.028 vs. 0.012, IQR 0.003-0.022, p=0.047).

**Conclusions:**

This study shows, that a sedentary behavior during SD might improve mood and slightly less impair the response time to auditory or visual stimuli than a higher activity level. Thus, PA could be an important modulator of clinical outcomes observed in individuals with affective disorders subjected to SD. PA should be accounted for in the SD protocols designed for future studies.

**Disclosure of Interest:**

None Declared

